# Children’s Arithmetic Strategy Use and Strategy Change from Grade 3 to Grade 4

**DOI:** 10.1007/s10763-025-10578-3

**Published:** 2025-05-29

**Authors:** Henning Sievert, Marian Hickendorff, Ann-Katrin van den Ham, Aiso Heinze

**Affiliations:** 1 University of Hildesheim, Samelsonplatz 1, Hildesheim, 31141 Germany; 2 Leiden University, Wassenaarseweg 52, Leiden, 2333AK Netherlands; 3 Hamburg University, Von-Melle-Park 8, Hamburg, 20146 Germany; 4 IPN - Leibniz Institute for Science and Mathematics Education, Olshausenstr. 62, Kiel, 24118 Germany

**Keywords:** Elementary school, Latent transition analysis, Mathematics, Strategy use

## Abstract

Children and adults use various strategies to solve arithmetic problems such as 701–698. Many studies have investigated students’ strategy use at a specific time point, but our knowledge about the longitudinal changes in students’ strategy use in elementary school is limited. Using a multilevel latent transition analysis, we examined individual differences in strategy use in multi-digit addition and subtraction problems of 1,947 students measured at the end of Grade 3 and the end of Grade 4. The analysis identified five different strategy use profiles, with the three largest profiles (mostly written algorithms, mostly mental computations, and a combination of written algorithms and mental computations) characterizing the strategy use of the large majority of students. For strategy use change over time, the analysis identified heterogeneous learning paths from Grade 3 to Grade 4. Nearly half of the students remained in the same strategy profile, but the other half moved to another profile. Students’ prior arithmetic knowledge and gender affect both strategy use in Grade 3 and strategy change between Grade 3 and 4, with amplifying effects. The classroom characteristics classroom intellectual composition and mathematics textbook had an effect on students’ strategy use in Grade 3, but not on strategy change. In conclusion, the current study shows how students differ not only in their arithmetic strategy use at one specific point in time but also in their learning paths, and that both student and classroom factors have impact.

**Supplementary Information:**

The online version contains supplementary material available at 10.1007/s10763-025-10578-3.

## Introduction

There are multiple ways to solve addition or subtraction problems like 701–698, such as indirect addition (how much to add to 698 to reach 701) or simplifying (adding 2 to both operands to obtain the easier problem 703–700; e.g., Torbeyns & Verschaffel, 2016). The acquisition of various strategies that can be applied insightfully, efficiently, and flexibly has become a major goal of elementary mathematics education worldwide (e.g., Baroody & Dowker 2003; Torbeyns & Verschaffel, 2016). Though both children and adults are known to use multiple strategies, we know little about individuals’ strategy change longitudinally, and what factors may play a role. These include individual factors like gender or prior arithmetical knowledge, but also classroom factors like curriculum materials or teacher qualification (e.g., Durkin et al., 2017; Fagginger Auer et al., 2016; McMullen et al., 2016). The current study’s aim is therefore to get more insight in elementary students’ arithmetic strategy use, their strategy use change trajectories, and factors that impact both aspects. Using a person-centered approach, latent transition analysis, on longitudinal strategy use data from a large-scale sample of students from Grade 3 and Grade 4, we utilized a unique opportunity to gain robust insights into students’ strategy use and strategy change. As such the findings can make a substantial contribution to our understanding of the acquisition and development of strategy competence and factors of importance, which can serve as a foundation to develop suitable opportunities to learn addition and subtraction strategies.

## Theoretical Background

### Multi-digit Addition and Subtraction Strategies

Solution strategies for arithmetic problems, are commonly categorized into number-based or digit-based (e.g., Verschaffel et al., 2009). The state curriculum in the German federal state Schleswig-Holstein, where our data were collected, prescribes one digit-based algorithm each for addition and subtraction (introduced in the second half of Grade 3) whereas number-based strategies are only referred to in general terms.^1^ Table 1 presents an overview of typical number-based strategies used in German elementary schools.

**Table 1:** Common number-based strategies for solving multi-digit addition and subtraction problems (illustrated by example problem 701–698 = 3; there are corresponding versions for addition problems, except for indirect addition)

Jump	Split	Compensation	Simplify	Indirect Addition
701–600 = 101 101–90 = 11 11–8 = 3	700–600 = 100 0–90 = –90 1–8 = –7 100–90–7= 3	701–700 = 1 1 + 2 = 3	703–700 = 3	698 + 3 = 701

Some teachers and textbooks avoid the split strategy in subtraction with borrowing to prevent intermediate negative results (e.g., –90, –7). These five strategies aren’t fully comprehensive, as students often invent or modify number-based strategies (e.g., Heinze et al., 2018; Hickendorff, 2020; Threlfall, 2002)

The jump and split strategies can be considered universal strategies, applicable in almost all cases for multi-digit addition and subtraction problems. By contrast, the compensation, simplify, and indirect addition strategies are particularly efficient for specific problem types (e.g., the example in Table 1; Heinze et al., 2018) since they may require fewer computation steps and/or no decompositions of the numbers given. These strategies are therefore also called shortcut strategies and are supposed to promote mental computation skills (e.g., Hickendorff, 2018). In the universal strategies, one number (jump) or both numbers (split) are decomposed, usually with regard to the place value system, in order to create simpler sub-steps. Compensation involves creating a simpler problem by rounding the subtrahend and then compensating back the difference in the second step. For simplifying, both numbers are changed in the same (subtraction) or opposite (addition) direction in order to generate a simpler problem. Finally, indirect addition utilizes the complementarity of addition and subtraction, by determining how much has to be added to the subtrahend to reach the minuend.

Stimulating number-based strategies and mental arithmetic is considered important for several reasons: It is not only useful in informal contexts and adult life, but also important for developing understanding of the place-value system, enhancing successfully learning and understanding the digit-based written algorithms, and promoting flexible strategy use and problem-solving skills (Threlfall, 2002; Torbeyns & Verschaffel, 2016). Therefore, the mathematics curriculum pays ample attention to number-based strategies before the introduction of written algorithms. Empirical research, however, repeatedly showed that many students, especially low and medium achievers, tend to use very few strategies or even only one “favorite strategy” (Csíkos, 2016; Heinze et al., 2009, 2018; Hickendorff, 2020; Selter, 2001; Torbeyns et al., 2017). Moreover, after they have been taught the written algorithm, they tend to use this strategy on almost all problems (e.g., Selter, 2001; Torbeyns & Verschaffel, 2016).

The acquisition of strategies and their flexible use relies on the development of several knowledge components, and on instruction. Importantly, both procedural and conceptual knowledge play a role: students need a solid understanding of numbers and operations to effectively use strategies for solving arithmetic problems (e.g., Pittalis, 2023; Torbeyns et al., 2012; Verschaffel, 2024). For instance, stimulating knowledge of number structure and relations and their representations contributes to students’ strategic competence (Venkant et al. 2024). The instruction of strategies by the teacher, either explicit or more implicit, also plays a crucial role in students’ strategy use (Blöte et al., 2001; Heinze et al., 2018). Furthermore, the cognitive model of Lemaire and Siegler (1995) states that by practicing multiple and varying tasks, students become faster and more accurate in their strategies and replace informal, cumbersome strategies with more efficient ones.

Since strategies play an important role in the arithmetic curriculum and students’ conceptual and procedural knowledge develops over time, it is likely that students’ strategy use develops as well. Some studies use a cross-sectional design including students from different grades to analyze these changes (e.g., Torbeyns et al., 2017), generally showing that students in higher grades are more inclined to use written algorithms, but are also more inclined to varied and flexible strategy use. However, these cross-sectional designs do not give insights into individual students’ strategy development, for which longitudinal data are needed. Some studies did collect strategy data for addition and subtraction at multiple time points (Heinze et al., 2018; Selter, 2001) but their analyses did not allow tracing individual students’ strategy use change, in other words: which students changed their strategy use and how? Schulz and Leuders (2018) did address this question in the domain of multi-digit division, using a person-centered approach (latent transition analysis) on a longitudinal data set with 208 German fourth graders. They found different strategy profiles and different learning trajectories, as well as relations with student factors. The current study uses the same approach to gain insights in individual differences in students’ strategy change in the domain of multi-digit addition and subtraction.

### Factors Related to Strategy Use for Arithmetic Problems in Elementary School

Many efforts have been made to analyze possible factors affecting students’ strategy use, showing that students’ characteristics but also instructional factors like the textbook used can impact students’ strategy use (e.g., Durkin, 2017; Fagginger Auer et al., 2016; McMullen et al., 2016; Pittalis, 2023; Verschaffel, 2024). Regarding individual factors, students’ prior knowledge is important (e.g., Verschaffel, 2024). Students with higher (procedural and conceptual) prior knowledge tend to use strategies more effectively (e.g., Hickendorff et al., 2009, 2010), have a larger strategy repertoire (Hickendorff, 2020; Liu et al., 2018), are more inclined to use non-standard, innovative strategies and show more flexible and adaptive strategy use (e.g., Hickendorff, 2018; Schneider et al., 2011; Star et al., 2022; Torbeyns et al., 2006, 2017).

Another relevant individual characteristic is gender. Gender differences in strategy use have been reported throughout the school age, with girls showing a greater reliance on rules and procedures whereas boys seem to use more intuitive strategies (Carr & Davis, 2001; Carr & Jessup, 1997; Gallagher et al., 2000; Timmermans et al., 2007). In previous studies in multi-digit multiplication and division, we found that boys are more likely than girls to use mental computation, non-algorithmic strategies, or efficient shortcut strategies, whereas girls are more likely than boys to use standard algorithms (Fagginger Auer et al., 2016; Hickendorff, 2018). Suggested potential explanatory mechanisms include boys’ higher self-confidence in mathematics (Mejía-Rodríguez et al., 2021).

Regarding instructional factors, textbooks as intended curriculum and teaching activities as enacted curriculum were found to be associated with Dutch sixth graders’ strategy profiles in multiplication and division (Fagginger Auer et al., 2016). Relatedly, Sievert et al. (2019) showed that differences in textbook quality of strategy instruction affect German third graders’ strategy use in addition and subtraction problems.^2^ Hence, mathematics textbooks are a relevant factor to consider as predictor of students’ strategy use.

Strategy instruction does not only depend on the textbook: teachers, as designers of learning opportunities for strategy use, play a crucial role (e.g., Verschaffel et al., 2009). For instance, the way strategies are emphasized and dealt with in the classroom, explicitly and implicitly, affects students’ strategy use (Blöte et al., 2001; Durkin et al., 2017; Heinze et al., 2009, 2018).

In addition, several studies have demonstrated that a classroom’s overall performance level influences students’ mathematics achievement growth (e.g., Aucejo et al., 2022; Dar & Resh, 1986; Kiss, 2013). For instance, Kiss (2013) showed that elementary students benefit from having high-achieving peers, although high-achievers benefit less than middle and low-achievers. The composition of a classroom, in terms of students’ cognitive abilities, can affect instructional conditions (Aucejo et al., 2022), which might lead to a greater variety of student-invented strategies and solutions.

### Present Study

The present study aims to give insights in students’ individual differences in strategy use, in their changes in strategy use over time, and the role of individual and classroom factors. To capture these differences we used a person-centered approach: latent class analysis and its longitudinal extension, latent transition analysis (e.g., Hickendorff et al., 2018; Lanza & Cooper, 2016). These techniques identify unobserved subgroups of individuals by their response patterns – in the current study, profiles of the arithmetic strategies students used on the addition and subtraction problems.

The present study combines a large-scale sample with a one-year longitudinal design, which allows for the application of these advanced statistical models. Since extant research is mostly based on smaller samples and/or cross-sectional designs, the current study extends the state-of-the-art knowledge on students’ strategy use and change in three ways: (a) by charting individual differences in strategy profiles in a large sample of students who have been taught both number-based and digit-based strategies (i.e., at the end of Grade 3 in the current sample), (b) by identifying students’ individual differences in strategy use change in the course of Grade 4, and (c) by examining how both student and classroom characteristics are related to students’ strategy use profile and strategy change. Hence, in this study, we addressed the following research questions (RQ 1–3):

1. What profiles of strategy use in multi-digit addition and subtraction problems can be identified in students at the end of Grade 3?
2. What learning paths – reflected in different strategy change trajectories - can be identified between Grade 3 and 4?
3. How are students’ prior arithmetic knowledge and gender, and the classroom factors mean cognitive abilities, teacher qualification, and textbook used, related to students’ strategy profiles and strategy change?

## Method

### Participants and Design

This study is a secondary analysis of a four-year longitudinal dataset including 1,947 students (916 girls, 955 boys, 76 missing gender information) from 109 classrooms across 39 German schools (van den Ham & Heinze, 2022). We analyzed data from the measurements in Grade 3 and 4. The goal of this longitudinal study was the evaluation of a mathematics support program for low-achieving students in Grade 1 and 2, with two treatment groups (*n* = 958 and *n* = 514, respectively) and one control group (*n* = 475). In both treatment groups, additional teaching materials on basic arithmetic concepts (e.g., cardinal numbers, place value, and basic arithmetic operations) were provided, and teachers in the first treatment group were supported with two extra teacher working hours per week. For the current study we used tasks from the follow-up measures administered in Grades 3 and 4 (typically 9–10 years old in Germany). Since the teacher support program ended after Grade 2 and did not include advanced topics like multi-digit arithmetic strategies, we did not expect effects of the intervention on students’ strategy use in arithmetic in Grade 3–4. This expectation was supported by a non-significant effect of treatment versus control condition on the strategy profiles in Grade 3 and their transitions through Grade 4 (see Section “Student and Classroom Predictors (RQ 3)”). Therefore, the intervention was discarded in the current analyses.

Schools were selected by an education authority in Schleswig-Holstein from urban and rural areas to ensure a socio-economically and culturally diverse sample, representing about 10% of the state cohort. All teachers followed a uniform curriculum, emphasizing ‘different number-based strategies’ and one-digit algorithm for addition and subtraction. About 59% of teachers had studied mathematics during their teacher training, and most classrooms used one of four textbook series.^3^ The education authority conducted test administration, overseen by the state’s Data-Protection Supervisor. Test administrators were employed by the ministry and were trained by the Department of Mathematics Education of the IPN - Leibniz Institute for Science and Mathematics Education, Kiel, for each test. Hence, all tests were administered according to a standardized test manual, all data we received was fully pseudonymized, and no direct contact with students or teachers occurred.

### Measures

#### Strategy Use

The strategies used were inferred from students’ written solutions in the tests administered at the end of Grade 3 and Grade 4, respectively. The trained test administrators conducted the tests in a 30-minute session. The test contained different types of arithmetic problems, the arithmetic subtest assessing the strategy use allowed about one minute per item. In our strategy analysis, we focused on five categories that were encountered in students’ solutions: written algorithm, mental computation, jump strategy, split strategy, and other number-based strategies. The written algorithm category involved the use of written addition or subtraction algorithms, representing digit-based computations. The mental computation category included solutions without any notation of computation steps or partial work, such as jottings. For the number-based strategies (jump strategy, split strategy, and other number-based strategies), we assigned approaches that included jottings or partial or full computations. Other number-based strategies included the strategies compensation, simplify, indirect addition and mixtures of several number-based strategies.

**Fig. 1:**
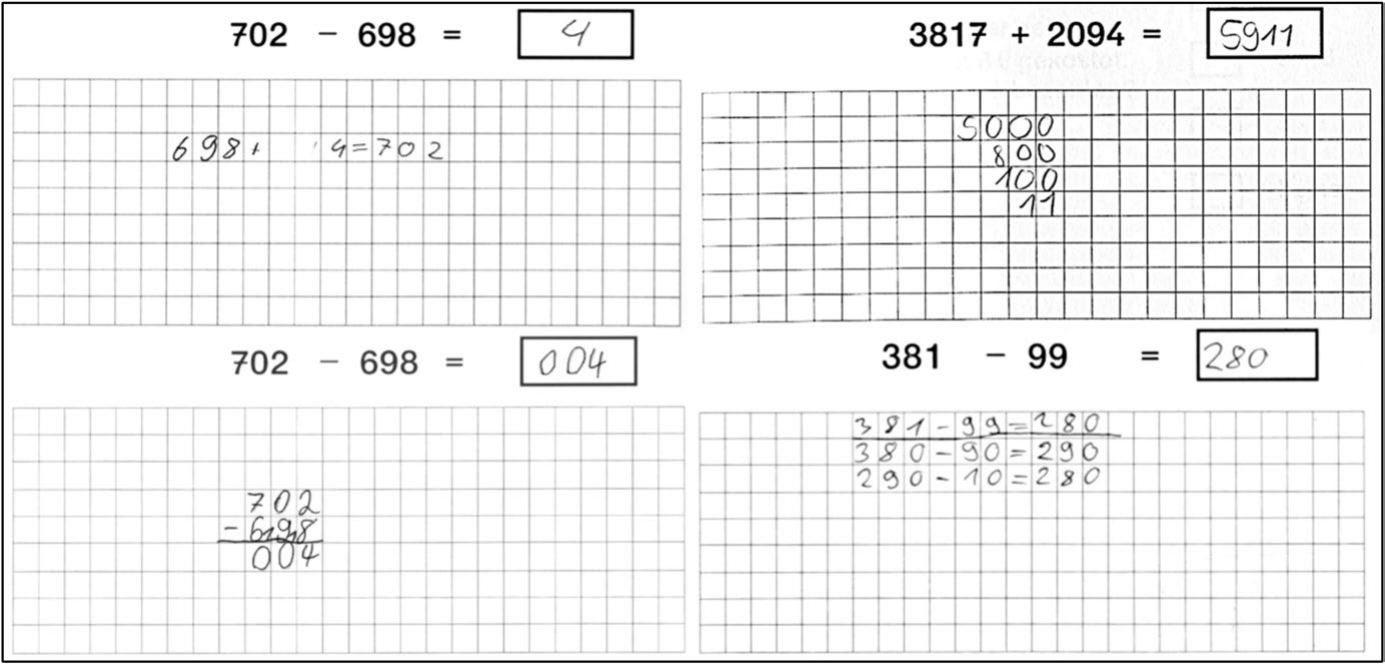
Examples of students’ strategies (top left: indirect addition, top right: split strategy (jottings), bottom left: written algorithm, bottom right: category “other number based strategies” (mixture of split and jump strategy))

These strategy categories were used to deductively classify students’ approaches to five problems (two addition and three subtraction) assessed at the end of Grade 3 and Grade 4. The problems were (A) 473+398, (B) 3817+2094, (C) 381–99, (D) 702–698, and (E) 7156–2478, and they were chosen to cover the range of strategies (i.e., from universal to task-specific strategies) discussed in Section “Multi-digit Addition and Subtraction Strategies”. Two problems ask for four-digit addition or subtraction. The problem (B) 3,817 + 2,094 can be solved, for example, by the task-specific strategies compensation (3,817 + 2,100 = 5,917 and 5,917–6 = 5,911) or simplifying (to the equivalent problem 3,811 + 2,100 = 5,911), whereas we expect universal strategies or written subtraction for the problem (E) 7,156–2,478. Students were asked to solve the problems in a way which they found fastest and most accurate. The answers were scored correct or incorrect, and the five-item scale had satisfactory internal consistency (Cronbach’s α =.71 and.72 in Grade 3 and Grade 4, respectively). The solutions were coded by eight trained research assistants. A random sample of 22% of the solutions was double-coded, providing a sufficient interrater agreement for each of the five problems (Cohen‘s κ:.83–.90). Deviations in the classification mainly occurred within the category “other number-based strategies”, as some examples cannot be clearly assigned. An example is shown in Fig. 1 (bottom right). The results showed that students used various strategies to solve these problems. Figure 1 illustrates four examples of student solutions.

#### Individual and Classroom Predictors

Our dataset includes two relevant student characteristics: gender and prior arithmetic knowledge. The latter was assessed by a Grade 3 arithmetic test adapted from Lipowsky et al. (2011)^4^ We used Item Response Theory ability score estimates, which has satisfactory reliability (WLE Person separation reliability:.94, indicating that the test discriminates between different ability levels very well). Classroom-level factors included teacher qualification (mathematics studied or not), intellectual composition (measured by cognitive abilities at school entrance – CFT 1-R, Weiß & Osterland, 2013), and the mathematics textbook used. The textbook variable had five categories (Textbook A-D, and others/no textbook). Textbook D was previously noted for high-quality number-based strategy instruction, while Textbook B ranked lowest (Sievert et al., 2019). In our analysis, we treated the textbook variable categorically, not using the quality scale due to its exclusion of written algorithms, crucial to this study. Including the “other textbooks or no textbook” category was necessary to maintain an adequate sample size.

### Data Analysis

With person-centered approaches latent class analysis (LCA) and its longitudinal extension latent transition analysis (LTA; e.g., Hickendorff et al., 2018; Lanza & Cooper, 2016), we aim to identify strategy use profiles and trajectories of strategy change. Similar approaches have been employed in previous studies on arithmetic strategy use (e.g., Fagginer Auer et al., 2016; Schulz & Leuders, 2018). LCA and LTA aim to identify latent clusters of students using a particular pattern or profile of strategies across the five addition and subtraction problems (RQ1). LTA additionally includes the probabilities to transition between these profiles over time (RQ2).

Finally, LTA allows testing the association of predictor variables with strategy profile and strategy change (RQ 3). The predictor variables included were students’ prior arithmetic knowledge (continuous), students’ gender (binary), teacher qualification (binary), classroom mean of general cognitive abilities (continuous), and the textbook used (nominal).

The analyses were conducted using Latent Gold 6.0, with ten categorical indicator variables: the strategy used on the five problems, at two time points (Grade 3 and 4). Model fit indices (BIC and CAIC) and conceptual appeal guided the selection of the number of classes or profiles. These indices represent a trade-off between model fit (log-likelihood) and model complexity (number of parameters): models with lower values are preferred. We employed multilevel LTA, since students are nested within classrooms and there may therefore be classroom effects, and we also had predictors at the classroom level.

## Results

### Profiles of Strategy Use (RQ 1)

Table 2 presents summary statistics of strategy use and accuracy, aggregated over the five problems, for both time points. The written algorithm is used more than 50% in both grades, with consistently high accuracy. Mental computation, utilized frequently, shows an increase in both frequency and accuracy between time points. In contrast, split and jump strategies are infrequent and decrease from Grade 3 to Grade 4.

**Table 2:** Use of the five strategies in percentages, and mean proportion correct per strategy (between brackets), aggregated over the five problems

	Grade 3	Grade 4
Written algorithm	55.7% (.71)	55.0% (.74)
Mental computation	33.0% (.48)	39.8% (.61)
Split strategy	5.1% (.50)	2.7% (.37)
Jump strategy	2.3% (.53)	0.5% (.65)
Other number-based strategies	3.9% (.21)	2.0% (.28)
Total	100% (.60)	100% (.67)

To identify individual differences in strategy use (RQ1) and strategy change (RQ2), we estimated several models. First, we ran latent class analyses (LCAs) for Grade 3 and Grade 4 separately. According to the BIC and CAIC, for Grade 3 five clusters were optimal, and for Grade 4 four clusters. Since the strategy use profiles from these separate LCAs were very similar, latent transition analysis (LTA) on the combined data from Grade 3 and 4 was justified. Fit indices suggested five clusters, and this model described students’ strategy use quite well. Including a multilevel component, accounting for students being nested into classrooms, further improved model fit. Details about the determination of the LTA clusters are given in the online Supplementary Materials.

**Fig. 2:**
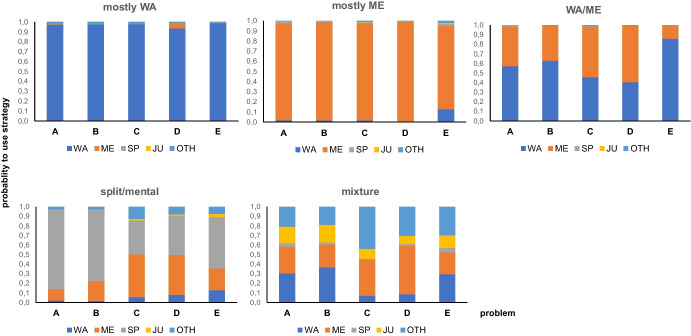
The five strategy use profile clusters from the latent transition analysis

Figure 2 shows the strategy use profiles in the five clusters. The first cluster shows a high likelihood (>.90) of using written algorithms across the five problems A-E and was therefore labelled *mostly written algorithms*. This profile characterized 47.6% of the students in Grade 3 and 38.7% in Grade 4.

Students in the second cluster had a high probability to use mental computations across all problems (>.95 on the problems A-D and .82 on problem E). This cluster was therefore labelled *mostly mental computations*. This profile characterized 25.0% of the students in Grade 3 and 28.0% in Grade 4.

The third cluster showed substantive probabilities of written algorithms and mental computation, which depended on the problem, most obvious for the most challenging problem E (7156–2478). Notably, this problem evokes a slightly different strategy use than the other problems in almost all clusters. We labelled this cluster *written algorithms and mental computations*. This profile characterized 17.1% of the students in Grade 3 and 29.3% in Grade 4.

Students in the fourth cluster had a high probability to use the split strategy on the two addition problems and to use split or mental computation on the three subtraction problems. We therefore labelled it *split/mental* (mixture of split strategy and mental computations). This profile characterized 4.7% of the students in Grade 3 and 3.0% in Grade 4.

Finally, the fifth profile is characterized by substantial probabilities for all strategies and was therefore labelled *mixture* (mixture of strategies). This profile characterized 5.7% of the students in Grade 3 and 1.7% in Grade 4.

Table 3 shows the mean performance on the five arithmetic problems for each strategy profile, where students were assigned to the cluster for which they had the highest posterior probability (modal assignment). The clusters differed in the proportion correct in Grade 3 (*F*(4,1427) = 58.822, *p* <.001) and in Grade 4 (*F*(4,1635) = 34.808, *p* <.001). Post-hoc analyses with Sidak correction showed that in Grade 3 all pair-wise differences were significant except between the *mostly mental computations*, s*plit/mental*, and *mixture* profiles. Post-hoc analyses in Grade 4 showed that all pair-wise differences were significant except between the *mostly written algorithms* and *written algorithms and mental computations* profiles, and between the *mostly mental computations* and *mixture* profiles. Overall, students who mostly used written algorithms performed best, followed by students who combined written algorithms and mental computations, and then students who mostly used mental computations or a mixture of strategies.

**Table 3:** Mean proportion correct on the five problems per strategy profile

Strategy profile	Grade 3 M (SD)	Grade 4 M (SD)
Mostly written algorithms	.71 (.271)	.74 (.261)
Mostly mental computations	.46 (.311)	.59 (.298)
Written algorithms and mental computations	.64 (.271)	.71 (.252)
Split strategy and mental computations	.51 (.316)	.44 (.296)
Mixture of strategies	.44 (.272)	.52 (.274)

### Transitions Between Strategy Profiles (RQ 2)

To characterize individual differences in students’ change in strategy use we interpreted the transition probabilities, that is, the probabilities to move from a certain strategy profile in Grade 3 to each of the profiles in Grade 4 (see Table 4). The *mostly written algorithms*, *mostly mental computations*, and *written algorithms and mental computations* profiles appear to be the most stable ones. However, since the probability to remain in each of these profiles ranged between .42 and .57 there is still substantial instability for each profile. Students in the *split/mental* profile were most likely to move to the *mostly mental computations* profile, while students in the *mixture* profile were most likely to move to the *written algorithms and mental computations* profile. Although students of this latter profile were most likely to remain in this profile, the probabilities to move to the *mostly written algorithms* or *mostly mental computations* profile are about the same (.28).

**Table 4:** Transition probabilities from Grade 3 to Grade 4

	To Grade 4				
From Grade 3	(1) Mostly written algorithms	(2) Mostly mental computations	(3) Written algorithms/mental comp.	(4) Split strategy/mental computations	(5) Mix of strategies
(1) Mostly written algorithms	**.57**	.14	.25	.02	.02
(2) Mostly mental computations	.19	**.53**	.24	.03	.00
(3) Written algorithms/mental computations	.28	.28	**.42**	.01	.00
(4) Split strategy/mental computations	.19	.46	.24	**.11**	.01
(5) Mixture of strategies	.19	.23	.37	.10	**.11**

**Fig. 3:**
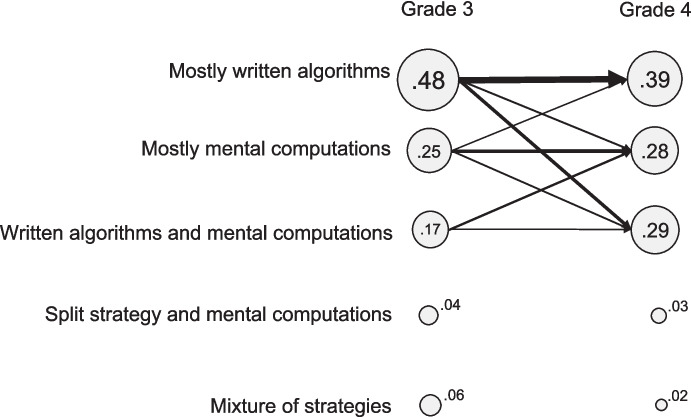
Graphical representation of five strategy use profile clusters and Grade 3 to Grade 4 transition probabilities (shown only for paths taken by at least 4% of the students)

Figure 3 provides a visual representation of the cluster sizes in Grade 3 and 4 and the transition probabilities. The areas of the circles reflect the size of the profiles, and the thickness of the arrows indicates the absolute percentage of students taking that path.

### Student and Classroom Predictors (RQ 3)

The five-cluster multilevel latent transition model was taken as the empty measurement model to which we added the effects of student and classroom predictors. We first tested the effect of teachers’ participation in the Grade 1–2 support program (the treatment conditions, merged into one category, vs. the control group, see Section “Participants and Design”). As expected, the support program has no effect on students’ strategy profile in Grade 3 (χ^2^ (4) = 8.953, *p* =.062) nor on the change in strategy use between Grade 3 and Grade 4 (χ^2^ (4) = 5.387, *p* =.25) and was therefore discarded from further analyses.

To answer RQ3, we first tested the effects of each predictor separately (see online Supplementary Materials). In these analyses, all five predictors showed significant effects on the strategy profile prevalences in Grade 3. For the transitions, only the effects of prior arithmetic knowledge, gender, and textbook were significant. Taking these results as a starting point, we ran analyses that included all predictor variables simultaneously, resulting in an effective sample size of 1293 students with complete predictor data. In the first set of models, we examined the effects of the predictors on the Grade 3 profile prevalences. All five predictors were included because of their significant bivariate relations with Grade 3 profile prevalences. In this simultaneous analysis, the effect of teacher qualification was no longer significant (*χ*^2^ (4) = 5.03, *p* =.28) and was thus removed from the model.

In the second set of models, we added predictors effects on the transition probabilities. The effects of students’ prior arithmetic knowledge and gender, classroom mean of cognitive abilities, and textbook on Grade 3 profile prevalences were retained in the model. Additionally, we included gender, prior arithmetic knowledge, and textbook as predictors of the transition probabilities because of their significant bivariate relations with the transitions. In this simultaneous analysis, the effect of textbook on the transition probabilities was no longer significant (*χ*^2^ (16) = 25.90, *p* =.055) and was therefore removed from the model.

**Fig. 4:**
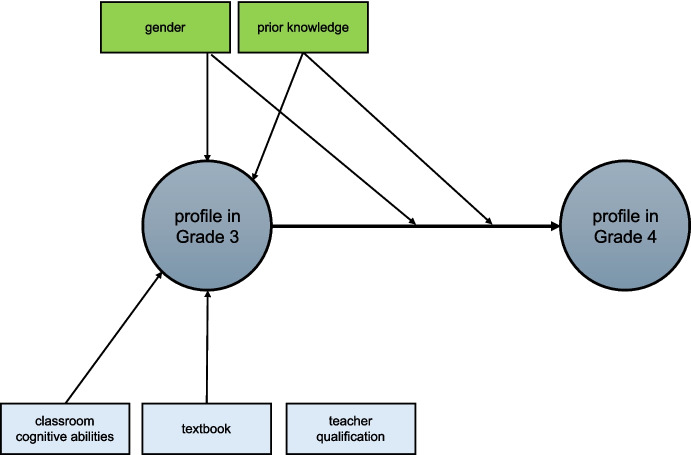
Final multilevel latent transition model with predictors for students’ strategy use in Grade 3 and strategy change between Grade 3 to Grade 4

In the final model (see Fig. 4), students’ prior arithmetic knowledge and gender had an effect on both strategy use in Grade 3 and strategy change between Grade 3 and 4, and the classroom characteristics classroom intellectual composition and mathematics textbook had an effect on students’ strategy use in Grade 3, but not on strategy change.

Below, we report and interpret the findings of the significant regression coefficients per predictor. The higher students’ arithmetic knowledge, the higher the likelihood to be in *written algorithms and mental computations* profile in Grade 3 (*β* = 0.166, *z* = 2.76, *p* =.006), and the lower the likelihood to be in *mostly mental computations* profile in Grade 3 (*β* = −0.140, *z* = −2.98, *p* =.003). Furthermore, students with higher arithmetic knowledge were more likely to move to the *written algorithms and mental computations* profile from Grade 3 to Grade 4 (*β* = 0.266, *z* = 3.56, *p* <.001), and less likely to move to the *mostly written algorithms* profile (*β* = −0.144, *z* = −2.05, *p* =.040).

Regarding gender, boys were more likely than girls to be in *mostly mental computations* profile (*β* = 0.369, *z* = 6.23, *p* <.001) and less likely than girls to be in *mostly written algorithms* profile (*β* = 0.315, *z* = 5.98, *p* <.001) in Grade 3. Furthermore, boys were also more likely than girls to move to the *mostly mental computations* profile (*β* = 0.402, *z* = 4.18, *p* <.001) and less likely than girls to move *to mostly written algorithms* profile (*β* = 0.249, *z* = 2.80, *p* =.005) from Grade 3 to Grade 4.

In classrooms with higher mean cognitive abilities students were less likely to be in *mostly written algorithms* profile (*β* = −0.060, *z* = −1.99, *p* <.001) and to be in *written algorithms and mental computations* profile (*β* = −0.097, *z* = −3.92, *p* <.001) in Grade 3.

Finally, there were some differences between textbooks in Grade 3 profile prevalences: Students with *Textbook C* were relatively likely to be in *mostly written algorithms* profile (*β* = 0.407, *z* = 3.13, *p* =.002) and in *written algorithms and mental computations* profile (*β* = 0.345, *z* = 2.00, *p* =.046). Students with *Textbook D* are relatively unlikely to be in *mostly written algorithms* profile (*β* = −0.320, *z* = −2.93, *p* =.003). Students with other or no textbook are relatively likely to be in *split/mental* profile (*β* = 0.508, *z* = 2.61, *p* =.009).

## Discussion

Using a novel longitudinal approach on a large-scale sample of students from Grade 3 and 4, we were able to capture individual differences in students’ strategy use (RQ 1) and strategy change (RQ 2), and relate that to student and classroom factors (RQ 3).

For RQ 1, we identified five profiles of strategy use. Students in the most prevalent profile very consistently used written algorithms on all problems. The two other quite prevalent profiles showed consistent use of mental computation, or combining written algorithms and mental computation, partly dependent on the problem. Finally, there were two rather small profiles: a mixture of the split strategy and mental computation and a mixture of all strategies. Including a multilevel component, accounting for students being nested into classrooms, improved model fit, suggesting an impact of classroom factors on students’ strategy use (addressed in RQ 3). Generally speaking, these results are in line with previous studies identifying strategy use profiles in multi-digit arithmetic, where most profiles include consistent strategy use across problems and a large preference for written algorithms (Fagginger Auer et al., 2016; Hickendorff et al., 2009; Torbeyns et al., 2017). Furthermore, the strategy profiles differed in performance: students who mostly used written algorithms performed best, followed by students combining written algorithms and mental computations, and then students mostly using mental computations or a mixture of strategies. Notably, these differences were less pronounced in Grade 4 than in Grade 3, suggesting students become more proficient in mental computations.

For RQ 2 findings showed different strategy change trajectories in the course of Grade 4. Students in the three largest profiles – mostly written algorithms, mostly mental computation, and combining written algorithms and mental computation – had probabilities to remain in their profile of around .50. Students in the two small profiles were most likely to move to one of the larger profiles, mostly mental computations or combining written algorithms with mental computation. Overall, slightly more than half of the students change strategy profile, with a general pattern of moving away from consistently using written algorithms to combining written algorithms with mental computations, and moving away from mixtures of (number-based) strategies towards written algorithms and/or mental computations. Note that analyzing only strategy frequencies (i.e., traditional, variable-centered analyses) would have obscured changes in strategy use patterns. For example, based on the strategy use frequencies one would conclude the use of the written algorithm is very stable (55 per cent) across the two grades. However, the person-centered LTA results show that the share of students who *consistently* use written algorithms across the problems decreases and the share of students who *combine* written algorithms with mental computation increases.

The instability of students’ strategy profiles underlines the development of students’ strategy use as prolonged process. This suggests that, although addition and subtraction strategies are predominantly covered in Grade 2 and 3 in German elementary schools, students’ actual strategy use develops further in Grade 4. Possible explanations for this continuing development include the extension of the number range from 1,000 (Grade 3) to 1,000,000 (Grade 4) in the curriculum for addition and subtraction, and the introduction of the written algorithm for multiplication, which includes digit-based addition. On the other hand, the finding that nearly half of the students remained in the same strategy profile suggests that earlier instruction is very significant and can affect future mathematical behavior.

At the end of Grade 4, the vast majority of students show profiles indicating only the use of written algorithms and/or mental computation. This decrease of written number-based strategies can be interpreted as an ongoing development of students’ number sense (Torbeyns et al., 2012), which is accompanied by an improvement of students’ speed and accuracy when practicing these strategies (Lemaire & Siegler, 1995) and allows more students to solve certain problems with purely mentally executed number-based strategies. This interpretation is supported by the higher performance with mental computation in Grade 4 than in Grade 3. Written algorithms are also expected to increase in speed and accuracy as they become more routine in the course of Grade 4, so they are used more frequently. Consequently, they might also be executed mentally on some tasks (e.g., Csíkos, 2016), which could explain that the accuracy of written algorithms did not increase.

Finally, in RQ 3 we addressed the role of student and classroom factors in students’ strategy use and strategy change over time. Our findings showed that the student factors prior arithmetic knowledge and gender affect both strategy use in Grade 3 and strategy change between Grade 3 and 4, whereas the classroom characteristics classroom intellectual composition and mathematics textbook had an effect on students’ strategy use in Grade 3 but not on strategy change in Grade 4. Teacher qualification showed no significant effects on strategy profiles.

First, students with higher prior arithmetic knowledge were more likely to already be in the combined written algorithms/mental computations profile at the end of Grade 3, as well as to move to this profile in the course of Grade 4. This is in line with the well-known influence of students’ prior arithmetic knowledge on the variability of strategy use (e.g., Hickendorff, 2020; Torbeyns et al., 2006; Verschaffel, 2024). In our test, some problems are not easily solved by mental computations (e.g., the four-digit number problems 3817+2094 and 7156–2478), whereas other problems like 381–99 or 702–698 can easily be solved mentally. The written algorithms/mental computation profile generally aligns with this, suggesting students in this profile take problem characteristics into account. Higher arithmetic knowledge likely enhances students’ competence to analyze the problems and developing or choosing an efficient strategy, such as a mentally-performed shortcut strategy. This interpretation is further supported by the findings that students with higher arithmetic knowledge were less likely in the mostly mental profile in Grade 3, and less likely to move to the consistent written algorithms profile in Grade 4. Conversely, students with lower arithmetic knowledge probably have fewer strategies available but also a smaller basis of knowledge about number relations when solving problems, which can explain why students with lower prior arithmetic knowledge are more likely to move to the consistent use of written algorithms profile during Grade 4.

Second, students’ gender affected both strategy use and strategy change. Boys were more likely than girls to be in the mental computation profile in Grade 3 and to move to this profile in the course of Grade 4, whereas girls were more likely to be in the written algorithms profile in Grade 3 and to move to this profile in the course of Grade 4. These gender differences are in line with previous research, showing girls are more inclined to use standard strategies (e.g., Hickendorff et al., 2010; Timmermans et al., 2007). Importantly, the current study shows that these gender differences amplified over the course of one year, suggesting that it is a persistent phenomenon that continues to develop, even when instruction has moved on to more advanced topics.

One explanation for the gender differences might be girls having a lower self-concept for mathematics and higher mathematics anxiety than boys (Dowker et al., 2016; Mejía-Rodríguez et al., 2021), which can lead to girls relying more on (proven) rules and procedures. For instance, a recent study showed that children with higher levels of mathematics anxiety were less likely to use advanced problem-solving strategies (Ramirez et al., 2016). Another explanation may be that boys weight accuracy and speed differently, with boys having a higher preference for fast-but-risky mental computation than girls (Fagginger Auer et al., 2018; Hickendorff et al., 2010).

Third, higher classroom level of general cognitive abilities resulted in a lower likelihood to consistently use written algorithms or to combine the use of written algorithms and mental computation. This might imply a higher use of number-based strategies, performed mentally or with written work. Conversely, it could also imply a higher rate of mentally performed algorithms, or a lower need to write down a strategy, due to higher self-confidence in this group. Generally speaking, this result fits the general finding that classroom intellectual composition influences students’ achievement growth in mathematics (e.g., Aucejo et al., 2022; Dar & Resh, 1986; Kiss, 2013).

Fourth, no effects of teacher qualification on strategy use profile prevalences in Grade 3, which is in line with for instance the German TIMSS 2019 findings, also showing no relation between teacher qualification in mathematics and students’ mathematics achievement (Mullis et al., 2020). This might suggest teacher qualification is confounded with other teacher or classroom characteristics.

Finally, we found mathematics textbooks affecting student’s strategy use in Grade 3, in line with findings from Fagginger Auer et al. (2016) and Sievert et al. (2019). These textbook differences seem to align with the rating of the textbooks’ quality of instruction of number-based strategies from Sievert et al. (2019). That is, students instructed by the lower-quality *Textbook B* – reflected in fewer presentations of number-based strategies and fewer learning opportunities to compare strategies – had a higher tendency to consistently use written algorithms. Conversely, students instructed with one of the higher-quality textbooks *Textbook D* or *Textbook A* were less likely to consistently use written algorithms and more likely to combine written algorithms with mental computation. This suggests that the textbook’s emphasis on number-based strategies affects strategy use, particularly of written algorithms. However, there were no robust textbook effects on students’ change in strategy use between Grade 3 and 4, probably because the strategy instruction for multi-digit addition and subtraction is mainly covered in Grade 2–3.

### Implications for Educational Practice

Empirical research shows, on the one hand, that the acquisition of arithmetic strategies and their flexible use is challenging for elementary students and, on the other hand, that students’ learning processes can be influenced by instruction (Blöte et al., 2001; Heinze et al., 2018; Hickendorff, 2020; Torbeyns et al., 2017). Our findings may help educators organize opportunities to learn addition and subtraction strategies, and imply several suggestions for the mathematics classroom to stimulate flexibility in students’ strategy use (Verschaffel, 2024).

First, our longitudinal findings suggest that prior knowledge is relevant not only for strategy acquisition, but also for a further development of strategy use. Since many strategies are based on a profound understanding of numbers, operations, and their relations, many strategies require solid conceptual knowledge. Conceptual and procedural knowledge have bi-directional relations (Rittle-Johnson et al., 2015), underlining the importance of addressing students’ conceptual knowledge on numbers and operations when teaching arithmetic strategies, and – vice versa – considering the meaning for later strategy competence when teaching numbers and operations in earlier grades.

Second, quite a few students consistently used mental computation to solve the multi-digit arithmetic problems, as has been found in multiplication and division as well (Fagginger Auer et al., 2016; Heinze et al., 2009; Hickendorff et al., 2009, 2010). This may be problematic, since mental computation less often leads to a correct answer, and students in the consistent mental computations profile performed worse than students using written algorithms consistently. Teachers should therefore be aware that this tendency exist, and that it may be related to valuing speed over accuracy (Hickendorff, 2020). It may thus be fruitful for teachers to promote and stimulate to write down their steps in number-based strategies – with special attention for boys – since this might foster internalization and improve accuracy of mental computations. Research in multi-digit division has shown that stimulating written strategies indeed increases performance (Fagginger Auer et al., 2018; Hickendorff et al., 2010).

On the other hand, there was more growth in performance from Grade 3 to Grade 4 with mental computation than with written algorithms, suggesting that students can become more proficient in computing the answer to the problems without writing anything down. Furthermore, quite a few students moved from consistent written algorithms to combining written algorithms with mental computation, particularly on the easier problems, suggesting they become better able in choosing between written algorithms and mental computations. At least as important as mastering the written algorithms may be to know when to use them and when another (number-based) strategy may be more appropriate. Teachers may stimulate such strategy flexibility by creating classroom discussion and reflection on the use of written algorithms versus other strategies.

Finally, textbook has an influence on students’ strategy use. According to the findings of Sievert et al. (2019), different features define textbook quality for strategy instruction: how strategies are offered and instructed, the frequency of these strategies, and the opportunities to contrast and compare strategies. These criteria are useful for teachers to evaluate their textbook, to supplement it where learning opportunities are limited, and to compensate for this with additional material, and should thus be implemented in teacher education and training. Furthermore, they are relevant for textbook authors when (re-)writing textbooks, and for policy makers when selecting and approving textbooks.

### Limitations

Though our analysis is based on a large and longitudinal dataset which includes a comprehensive set of variables, our study has several limitations. First, as the approach of this study was exploratory focusing on the actual and documentable use of strategies, we had to work with the strategies that could be inferred from students’ written work, and as such did not have information about the strategies used when students did not write anything down. Hence, the category “mental computation” may also contain guesses, or mentally executed standard algorithms. Other studies did investigate the strategies students in mental calculations. For instance, Csíkos (2016) reports rates of 27–39% (jump), 17–51% (split), 0–3% (simplifying), 7–9% (indirect addition) and 21–28% (written algorithm), depending on the problem and the operation given. These patterns suggest that in mental calculation students are relatively more likely to use number-based strategies and less likely to use the written algorithm.

A second limitation relates to the fact that we did secondary analyses on data collected for other purposes. Therefore, the selection of predictors is limited to the ones included in the dataset and identified by earlier research. For example, affective factors (e.g., students’ confidence and enjoyment of mathematics, beliefs, or implicit classroom norms) were not available and therefore have been not considered.

Third, although the curriculum and the textbooks used give indications about the instruction of strategies in the classrooms, we have no information about the actual classroom activities possibly influencing students’ strategy use, for instance, how much strategy use is emphasized and discussed after the introduction of the written algorithm.

Fourth, teacher qualification was scored binary, which does not contain much information about the qualification of out-of-field teachers. Depending on the year of examination and the federal state in which they studied, teachers’ qualification varies considerably. Hence, this group might not be homogeneous enough to yield clear effects. In addition, it is possible that the use and development of strategies as a specific topic has not been thouroughly addressed in the mathematics teacher education, so that subject teachers do not have sufficient professional knowledge either.

Fifth, the measure of strategy use in our study was limited to five problems, which could impact the reliability. However, students used the complete range of strategies to solve the five problems and previous studies showed similar effects while using low item numbers as well (e.g., Hickendorff et al., 2010; Torbeyns et al., 2017). Therefore, we are confident that the five problems are a reliable indicator of students’ strategy use.

Finally, the data for this study were collected in Germany and therefore depends on specific cultural conditions. Accordingly, the results may be transferable to other countries only to a limited extent. Nevertheless, the results can enrich the international state of research, as there are countries with comparable curricular requirements and a similar situation in terms of strategy use among elementary school students.

## Conclusion

The current study shows how students differ not only in their arithmetic strategy use at one specific point in time but also in their learning paths, and that both student and classroom factors play a role. The most important aspect of students’ strategy use was whether they used written algorithms (more common in girls) or mental computation (more common in boys), or combined the two, dependent on the problem (more common in students with higher prior knowledge). Students’ learning paths showed stability on the one hand, suggesting that instruction before the end of Grade 3 can have a profound effect on students’ future mathematical behavior. This is further supported by the finding that the textbooks’ quality of strategy instruction in Grade 2–3 plays a role in students’ strategy profile at the end of Grade 3. On the other hand, there was instability in students’ strategy use, with over half of the students changing strategy profile. This underlines the continuous development of students’ strategy use, even after instruction in these strategies. In this continuing development, differences related to students’ prior arithmetic knowledge and gender amplified. Teacher and educators should be aware of the nuanced process of strategy acquisition and development to strive for an optimal balance in acquiring and using different strategies, in all students.

## Supplementary Information

Supplementary Material 1.

Supplementary Material 2

## Data Availability

All data used were collected in accordance with relevant laws and in consideration of privacy guidelines, as well as with the approval of the relevant Ministry of Education. Due to these privacy guidelines and the fact that we carried out a secondary analysis of existing data collected for other purposes, we are not allowed to pass them to third parties.

## Footnotes

1 This holds at the time the data were collected. Recently, a new curriculum was published that gives specific examples of number-based strategies (jump, split, simplify, etc.), whereas the previous curriculum only referred to number-based strategies in general terms.

2 This study uses the same data base as Sievert et al. (2019). Whereas the former study examined strategies offered in textbooks and its relation to students’ use of efficient strategies in Grade 3, this present study exploratively analyzes patterns of strategy use in both Grade 3 and 4 as well as the change in strategy use over time, and the relations with predictors. In addition to a different perspective and focus (explorative, person-centered on actual strategy use), and thus a different category system of strategies, we also used a different subsample, two time points instead of one, allowing for an analysis of variations (Grade 3 and Grade 4), and a more elaborated methodology.

3 Elementary school teachers in Germany are generalists, trained in 2-3 subjects at university, so some graduated in mathematics while others did not.

4 Lipowsky et al. (2011) conducted a longitudinal study in higher-performing schools. Some difficult arithmetic items were replaced with easier ones in the test.
