# Supplementary material for: Children’s Arithmetic Strategy Use and Strategy Change from Grade 3 to Grade 4
Source: Int J Sci Math Educ. 2025 May 29;23(8):3577–98. doi: 10.1007/s10763-025-10578-3 (PMC12756257; doi:10.1007/s10763-025-10578-3)
Supplement: Supplementary file 1 — Supplementary Material 1. [file 10763_2025_10578_MOESM1_ESM.docx]

**Online Supplement 1 – Cluster model selection**

To identify individual differences in strategy use (RQ1) and strategy change (RQ2), we estimated several models. First, we ran latent class analyses (LCAs) for data from the two time points separately. According to the BIC and CAIC, for Grade 3 a model with five clusters was optimal and for Grade 4 a model with four clusters. Looking at the profiles of both LCAs shows that the first three profiles were very similar at the two points in time. The two remaining, small, profiles in Grade 3 are grouped together in one, even smaller, profile in Grade 4. Estimating a five-cluster model for Grade 4 results in very similar profiles as the five-cluster model for Grade 3. Since the strategy use profiles from separate LCAs for Grade 3 and Grade 4 were very similar, latent transition analysis (LTA) on the combined data from Grade 3 and 4 was justified.

As a first step the number of profiles had to be determined. To that end LTA-models with one to seven latent clusters were estimated (Table 3). Based on BIC and CAIC, models with five or six clusters were selected for further evaluation. In the six-cluster model, the two smallest clusters from the five-cluster model were re-distributed in three even smaller clusters which were hard to interpret and were thus not very meaningful. Therefore, the model with five clusters had the highest conceptual appeal, and it also fitted the data well: the mean classification error was .067, meaning that on average students are classified with 93.3 percent certainty, and the R^2^ entropy was .856, indicating that clusters are well-separated.

Next, we accounted for the multilevel structure of the data, which improved model fit. The resulting multilevel latent transition model described students’ strategy use quite well, with mean classification error of .058 (classroom level) and .068 (student level) and R^2^ entropy values of .745 (classroom level) and .856 (student level).

**Table 3.**

*Model fit statistics Latent Transition Analyses without multilevel component (lowest value of BIC and CAIC in boldface)*

| # clusters | LL | # par | BIC | CAIC | Classification error | R^2^ entropy |
| --- | --- | --- | --- | --- | --- | --- |
| 1 | -13898.7 | 20 | 27949.0 | 27969.0 | .000 | 1.00 |
| 2 | -10060.8 | 43 | 20447.3 | 20490.3 | .016 | .932 |
| 3 | -9456.9 | 68 | 19428.9 | 19496.9 | .023 | .920 |
| 4 | -9131.0 | 95 | 18981.6 | 19076.6 | .069 | .851 |
| 5 | -8955.9 | 124 | 18850.9 | **18974.9** | .068 | .856 |
| 6 | -8826.6 | 155 | **18827.2** | 18982.2 | .066 | .872 |
| 7 | -8739.3 | 188 | 18902.5 | 19090.5 | .075 | .856 |
