# Supplementary material for: Children’s Arithmetic Strategy Use and Strategy Change from Grade 3 to Grade 4
Source: Int J Sci Math Educ. 2025 May 29;23(8):3577–98. doi: 10.1007/s10763-025-10578-3 (PMC12756257; doi:10.1007/s10763-025-10578-3)
Supplement: Supplementary file 2 — Supplementary Material 2 [file 10763_2025_10578_MOESM2_ESM.docx]

**Online Supplement 2 – Separate analyses**

Preliminary to the simultaneous analysis, we tested each predictor separately in a run of three models: (a) re-run the empty model for cases without missing data on the predictor at stake, (b) add the effect of the predictor on the Grade-3 cluster sizes (profile prevalences), and (c) also add the effect of the predictor on the transition probabilities. Table 1 presents the model fit statistics and statistical testing of the effects. All student and classroom predictors showed significant effects on the strategy profile prevalences in Grade 3, whereas for the transitions, only the effects of prior arithmetic knowledge, gender, and textbook turn out to be significant.

**Table 1**

*Statistical tests of effects of predictor variables on the cluster sizes in Grade 3 and on the transition probabilities from Grade 3 to Grade 4*

|  | **LL** | **BIC** | **CAIC** | **par** | ***χ*^2^** | ***df*** | ***p*** |
| --- | --- | --- | --- | --- | --- | --- | --- |
| *Prior arithmetic knowledge* | | | | | | | |
| Empty model | -8488.25 | 17944.00 | 18073.00 | 129 |  |  |  |
| Gr3 cluster sizes | -8477.62 | 17952.74 | 18085.74 | 133 | 21.26 | 4 | <.001 |
| Gr3-Gr4 transitions | -8460.97 | 17949.44 | 18086.44 | 137 | 33.30 | 4 | <.001 |
| *Gender* | | | | | | | |
| Empty model | -8667.19 | 18306.3 | 18435.3 | 129 |  |  |  |
| Gr3 cluster sizes | -8611.71 | 18225.47 | 18358.47 | 133 | 110.97 | 4 | <.001 |
| Gr3-Gr4 transitions | -8585.62 | 18203.42 | 18340.42 | 137 | 52.18 | 4 | <.001 |
| *Teacher qualification* | | | | | | | |
| Empty model | -6705.56 | 14348.54 | 14477.54 | 129 |  |  |  |
| Gr3 cluster sizes | -6699.31 | 14365.11 | 14498.11 | 133 | 12.50 | 4 | 0.014 |
| Gr3-Gr4 transitions | -6697.08 | 14389.72 | 14526.72 | 137 | 4.46 | 4 | 0.35 |
| *Classroom mean cognitive abilities* | | | | | | | |
| Empty model | -8897.71 | 18772.46 | 18901.46 | 129 |  |  |  |
| Gr3 cluster sizes | -8891.32 | 18789.98 | 18922.98 | 133 | 12.78 | 4 | 0.012 |
| Gr3-Gr4 transitions | -8891.08 | 18819.8 | 18956.8 | 137 | 0.47 | 4 | 0.98 |
| *Textbook* | | | | | | | |
| Empty model | -8349.68 | 17668.35 | 17797.35 | 129 |  |  |  |
| Gr3 cluster sizes | -8334.52 | 17758.21 | 17903.21 | 145 | 30.33 | 16 | 0.016 |
| Gr3-Gr4 transitions | -8319.25 | 17847.85 | 18008.85 | 161 | 30.55 | 16 | 0.015 |

The higher students’ arithmetic level, the higher their likelihood to be in the *written algorithms and mental computations* profile in Grade 3 (*β* = 0.183, *z* = 3.23, *p* = .0013) and the lower their likelihood to be in the *mostly mental computations* profile (*β* = -0.099, *z* = -2.27, *p* = .023) or the *mixture* profile (*β* = -0.181, *z* = -2.17, *p* = .030). Furthermore, the higher students’ arithmetic level, the more likely they transition to the *written algorithms and mental computations* profile from Grade 3 to Grade 4 (*β* = 0.286, z = 3.89, *p* < .001).

In Grade 3, boys were less likely than girls to be in the *mostly written algorithms* profile (*β* = -0.564, *z* = -5.75, *p* < .001) and more likely than girls to be in the *mostly mental computations* profile (*β* = 0.729, *z* = 6.60, *p* < .001). Similarly, boys were also less likely than girls to move to the *mostly written algorithms* profile between Grade 3 and 4 (*β* = -0.418, *z* = -2.43, *p* = .015) and more likely than girls to move to the *mostly mental computations* profile (*β* = 0.868, *z* = 4.69, *p* < .001).

Students with teachers who studied mathematics were more likely to be in the *mostly written algorithms* profile in Grade 3 than students from out-of-field teachers (*β* = 0.212, *z* = 3.62, *p* < .001). Teacher qualification did not affect the transition probabilities.

The higher the classroom mean of students’ cognitive abilities, the higher the likelihood students were in the *split/mental* profile (*β* = 0.073, *z* = 2.21, *p* = .027) and the lower the likelihood to be in the *mostly written algorithms* profile (*β* = -0.067, *z* = -2.93, *p* = .003).

There were some differences between textbooks in Grade 3: students using *Textbook B* were relatively likely to be in the *mostly written algorithms* profile (*β* = 0.327, *z* = 2.49, *p* = .013) whereas students using *Textbook D* (*β* = -0.311, *z* = -2.78, *p* = .005) or another/no textbook (*β* = -0.249, *z* = -2.08, *p* = .038) were relatively unlikely to be in the *mostly written algorithms* profile. Students using *Textbook A* were relatively likely to be in the *written algorithms and mental computations* profile (*β* = 0.327, *z* = 2.49, *p* =.013). Finally, students using another or no textbook were relatively likely to be in the *split/mental* profile (*β* = 0.549, *z* = 2.93, *p* = .003). For the transitions between Grade 3 and 4: although the omnibus test of textbook differences in transition probabilities was significant (Table 7) none of the individual coefficients reached statistical significance, |z| < 1.48. Hence, there are no reliable textbook variations in students' strategy change paths to report.
